# Late relapse of chronic myeloid leukemia after allogeneic bone marrow transplantation points to KANSARL (KANSL1::ARL17A) alteration: a case report with insights on the molecular landscape

**DOI:** 10.1007/s00277-024-05649-4

**Published:** 2024-02-07

**Authors:** Diletta Fontana, Giovanni Paolo Maria Zambrotta, Antonio Scannella, Rocco Piazza, Carlo Gambacorti-Passerini

**Affiliations:** 1 Department of Medicine and Surgery, University of Milano-Bicocca, via Cadore 48, Monza, MB 20900 Italy; 2 Hematology Division and Bone Marrow Unit, IRCCS, San Gerardo dei Tintori, Monza, Italy

**Keywords:** CML, Next-generation sequencing, Late relapse, KANSARL, BCR::ABL1

## Abstract

Chronic myeloid leukemia is a myeloproliferative neoplasm characterized by the presence of the Philadelphia chromosome and the consequent BCR::ABL1 oncoprotein. In the era before the introduction of tyrosine kinase inhibitors (TKIs), the only potentially curative treatment was allogeneic hematopoietic stem cell transplantation (HSCT). Here, we present the case of a patient affected by CML who experienced a relapse 20 years after allogeneic HSCT. Following relapse, the patient was treated with imatinib and bosutinib, resulting in a deep molecular response and successfully discontinued treatment. Additional analysis including whole-exome sequencing and RNA sequencing provided some insights on the molecular mechanisms of the relapse: the identification of the fusion transcript *KANSL1::ARL17A* (*KANSARL)*, a cancer predisposition fusion gene, could justify a condition of genomic instability which may be associated with the onset and/or probably the late relapse of his CML.

## Introduction

Allogeneic hematopoietic stem cell transplantation (HSCT) represented the only potentially curative therapy for chronic myeloid leukemia (CML) until 2000. Its application to CML patients showed a continuous decrease since the introduction of tyrosine kinase inhibitors (TKIs), which have transformed CML from a once fatal to a manageable disease for the vast majority of patients [1]. Indeed, in the pre-TKI era, allogeneic HSCT was the standard first-line therapy for CML patients, while now [2], it is reserved for patients who do not achieve a sustained cytogenetic remission or have progressive disease [3–6]. Large number of patients have undergone allogeneic HSCT since the early 1980s, and although most of the risk of death occurs within the first 2 years after transplantation, patients have an increased risk of mortality compared to the general population for at least 10 years post-transplant [7, 8]. The increased mortality is related to complications of chronic graft versus host disease (GVHD), infectious, second malignancies, organ dysfunction, and in some cases, relapse [9]. Usually, relapse after allogeneic HSCT occurs within the first years after transplant, but later relapses have also been described [9–12].

Here, we present the case of an Italian CML patient with Caucasian ethnicity, who experienced a relapse 20 years after allogeneic HSCT, performed in first chronic phase (CP). NGS analysis, including whole-exome sequencing (WES) performed on both the diagnosis and the relapse samples, and RNA sequencing of diagnosis sample, offer an intriguing insight into the pathogenesis of his relapse.

## Case presentation

The patient was a previously healthy 36-year-old man, who had been diagnosed with CML in CP in November 1996. Cytogenetics revealed 46,XY,t(9;22)(q34;q11) in 20 out of 20 metaphases. After treatment with hydroxyurea and interferon, in January 1997, he achieved complete hematological remission and he underwent allogeneic HSCT from a human leukocyte antigen (HLA)-identical sibling donor (sister). Conditioning regimen consisted in cyclophosphamide and total body irradiation (TBI), which caused hypothyroidism and sterility. Post-transplant follow-up was always negative for disease recurrence, and in 2001, it was interrupted due to the patient’s request.

In October 2016, he was admitted with symptoms of dyspnea, fatigue, dizziness, and weight loss. The peripheral blood examination revealed a severe anemia (Hb 79 g/L), a platelet count of 81 × 10^9^/L and a white blood cell (WBC) count of 3.8 × 10^9^/L, with 1.7 × 10^9^/L neutrophils (44.7%), 0.02 × 10^9^/L basophils (0.5%), some immature myeloid cells (1.8% myelocytes), 0.9% blasts and some pseudo-Pelger elements, thus fulfilling criteria for an accelerate phase CML. The physical exam was normal. Bone marrow (BM) aspiration and biopsy revealed a hypercellular marrow at 90% with myeloid hyperplasia and a shift toward immaturity, increased atypical megakaryocytes, and increased blasts (12%). Cytogenetics revealed 46,XX in 1 of 20 metaphases and 46,XY, + 8,t(9;22)(q34;q11),-17,der(18)t(17;18)(q11;p11) or -18,der(17),t(17;18)(p11;q11) in the remaining 19 metaphases. The presence of the *BCR::ABL1* transcript was confirmed by reverse transcriptase polymerase chain reaction (RT-PCR). In November 2016, imatinib treatment was started at a dose of 600 mg daily. After 6 months (in May 2017), the patient achieved a complete cytogenetic response (CCyR), with standard cytogenetic demonstrating 46,XX in all 20 metaphases, and a *BCR::ABL1*/*ABL1* RT-PCR ratio of 0.016% IS (International Scale), corresponding to a major molecular response (MMR, or MR3 = %IS ≤ 0.1). At 9 months (in August 2017), he achieved a deep molecular response (DMR) of MR5 (%IS ≤ 0.001). Subsequently, owing to intolerance to imatinib (particularly because of anemia-related symptoms, as well as lower limbs arthromyalgia and muscle cramps), the drug was firstly reduced to 400 mg daily for a brief period and then, in February 2018, the treatment was switched to bosutinib, at a starting dose of 300 mg daily, then lowered to 200 mg. The patient kept maintaining an optimal molecular response on subsequent monitoring, with an undetectable transcript level (MR5 = %IS ≤ 0.001) on RT-PCR since July 2018. Similar to imatinib, treatment with bosutinib was not well tolerated, owing to severe asthenia and lower limbs arthralgias. Therefore, TKI treatment was discontinued at patient’s request in October 2019, after 26 months of stable DMR, 14 months of stably undetectable transcript levels, and after 35 months of total TKI treatment duration. In March 2020, 5 months after bosutinib discontinuation, the RT-PCR analysis turned positive (*BCR::ABL1*/*ABL1* RT-PCR ratio of 0.004% IS; corresponding to MR4 = %IS ≤ 0.01), but the patient never lost MMR, with subsequent assessments ranging between MR3 and MR4 until the most recent follow-up, 43 months later (on May 2023).

## Materials and methods

### Patient’s samples

The patient provided written informed consent; this study was conducted in accordance with the Declaration of Helsinki. BM samples were collected at diagnosis and relapse, and leukemic cells were obtained by separation on a Ficoll-Paque Plus gradient (GE Healthcare). Surface markers were evaluated by fluorescence-activated cell sorting (FACS) analysis, and myeloid cells (positive for CD33, CD13, or CD117 staining) made up > 80% of the total cells.

### Whole-exome sequencing

WES was performed on paired bone marrow samples obtained at diagnosis and at relapse. Genomic DNA (gDNA) was extracted from 10 million WBC with PureLink Genomic DNA kit (Thermo-Fisher-Scientific) according to manufacturer’s instructions. Then, 1 μg of gDNA was used to generate exome libraries (Galseq). The Illumina Nextera® Rapid Capture Exome Kit (Illumina Inc.) was used to enrich the genomic libraries for the exonic regions. WES was performed with a mean coverage of 80X. Image processing and basecall were performed using the Illumina Real Time Analysis Software. Paired Fastq files were aligned to the human reference genome (GRCh38/hg38) using the BWA-MEM algorithm [13]. Duplicates were annotated using Samblaster. Quality of the aligned reads, duplicate removal, somatic variants calling, annotation, and copy number analysis were performed using CEQer2 [14], a graphical tool for copy number alteration (CNA) detection in the context of exome-sequencing experiments. Variants were annotated using ClinVar, dbSNP, ExAC, OncoScore, Polyphen2 HVAR, LRT, MutationTaster, MutationAssessor, FATHMM, PROVEAN, VEST3, CADD, DANN, MetaSVM, MetaLR, Integrated fitCons, GERP +  + , PhyloP7way Vertebrate, PhyloP20way Mammalian, PhastCons7way Vertebrate, and PhastCons20way Mammalian. Splicing variants were analyzed using SpliceFinder [15].

### RNA sequencing

Ten million cells were lysed in TRIzol (Thermo Fisher Scientific) and RNA was extracted according to manufacturer’s instructions. Then, 2 μg of RNA (concentration 400 ng/μl) were used for library preparation (Galseq); the average per-sample read count was 35 M. The library was sequenced on an Illumina HiSeq 2500 with 76 bp paired-end reads. FastQ sequences were aligned to the human genome (GRCh38/hg38) using Star [16] and processed with Samtools [17]. Bam files were analyzed using CEQer2, an evolution and integration of FusionAnalyser [18] and CEQer [14].

## Results

### Whole-exome sequencing analysis

Whole-exome sequencing analyses performed on diagnosis and relapse samples showed great matchability and superimposition between them. This evidence has led us to exclude the possibility of a new leukemia, independent of the first, originating from the transplanted cell (sister’s female genome), and thus confirming that it is in all respects a relapse.

### Changes in copy number alterations/chromosomal alterations between diagnosis and relapse

**Fig. 1:**
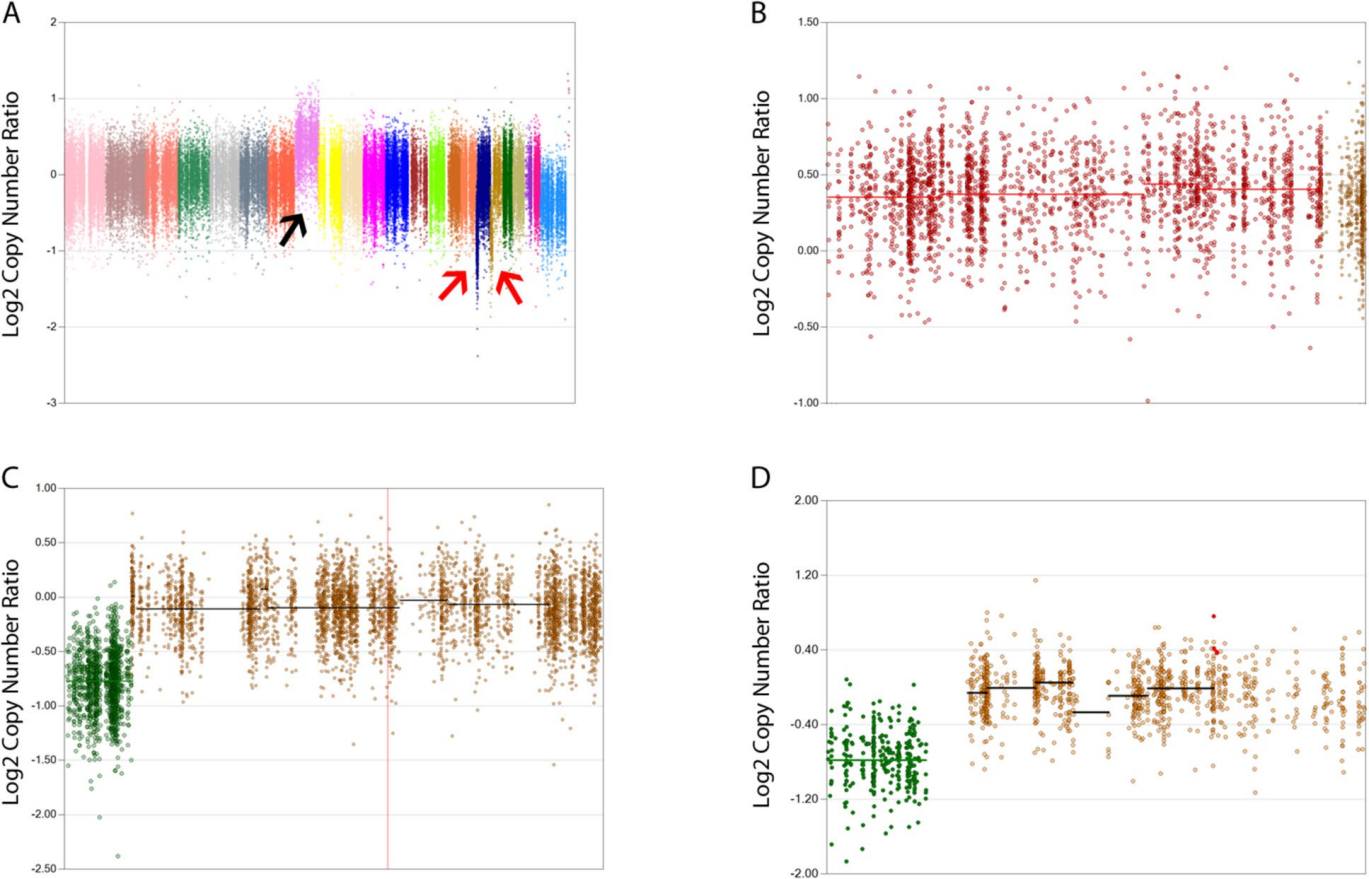
**A** Copy number plot (relapse vs. diagnosis). The black arrow points to an amplification of the whole chromosome 8; the red arrows point to deletions occurring in chromosomes 17 and 18. **B** Individual view of chromosome 8 (relapse vs diagnosis). Thick red horizontal bar identifies copy gain region. **C** Individual view of chromosome 17 (relapse vs. diagnosis). Thick green horizontal bar identifies copy loss region. **D** Individual view of chromosome 18 (relapse vs. diagnosis). Thick green horizontal bar identifies copy loss region

Our patient showed several changes in the pattern of CNAs from diagnosis to relapse (Fig. 1A). The paired diagnostic/relapse samples highlighted the presence of chromosome 8 trisomy (Fig. 1B) thus confirming cytogenetic results, isochromosome 17q, with loss of p53 (Fig. 1C), and a further deletion of a small portion of chromosome 18p (Fig. 1D). While + 8 and i(17q) copy number abnormalities are frequently found in CML patients [19] and, according to the WHO 2016 criteria, are “major route” abnormalities that define accelerated phase (AP) if found in *BCR::ABL1*^+^ cells at diagnosis [20], del(18p) is rarely detected.

**Fig. 2:**
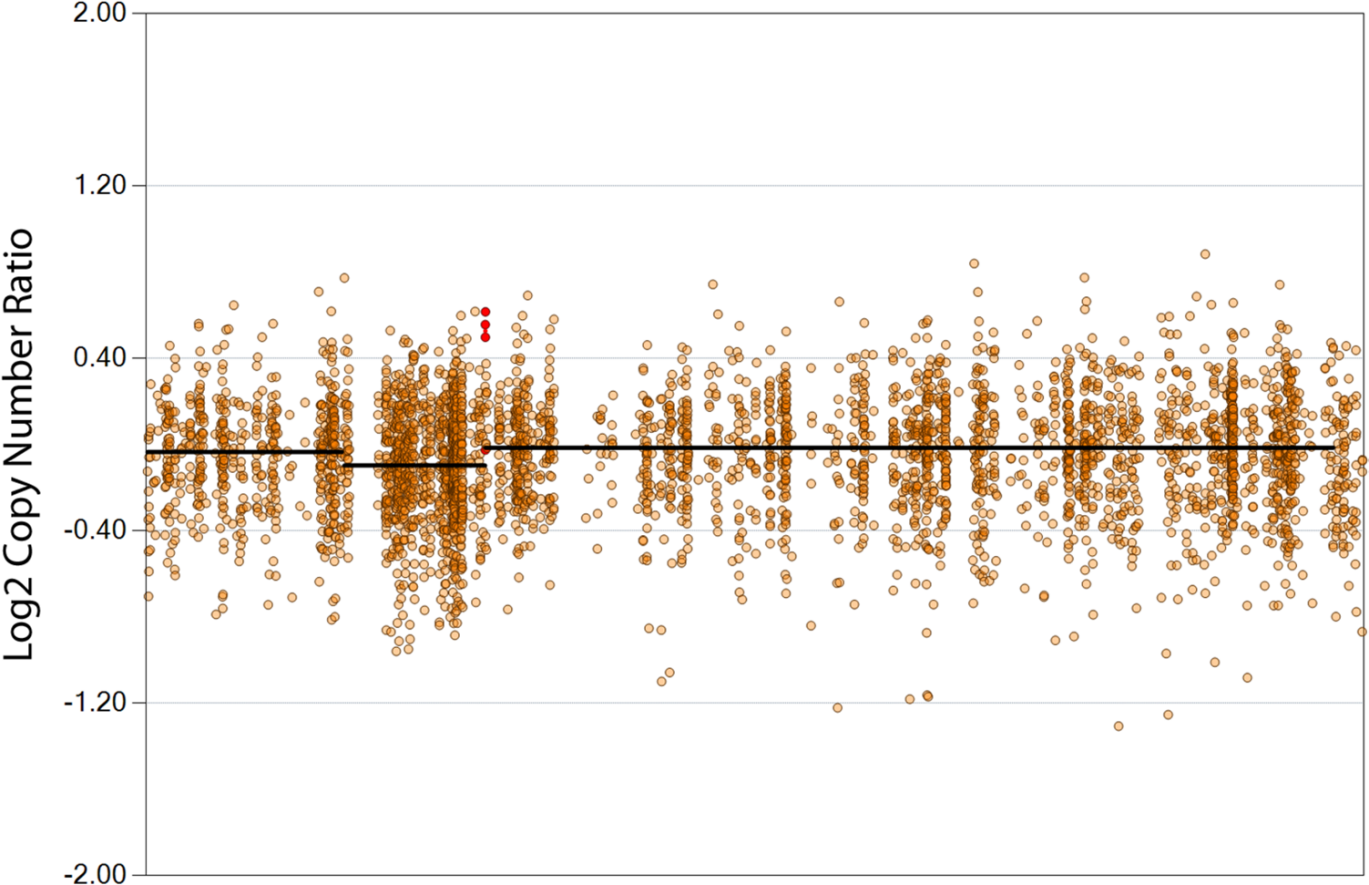
Individual view of chromosome 6 (relapse vs. diagnosis). No copy number alterations in genes encoding HLA complex are found

A known cause of CML relapse after HSCT, especially in case of haploidentical transplantation, is loss of heterozygosity of class 1 HLA, located on chromosome 6. This is due to the loss of the immune control effect exerted by donor T lymphocytes on the leukemic cells possibly surviving the conditioning regimen (the so-called graft versus leukemia (GVL) effect) [21]. Although at time of relapse our patient presented important copy number variations, particularly of genes on chromosome 8, 17, and 18, we did not find copy number variations involving genes on chromosome 6, where genes encoding HLA complex are located (Fig. 2). This fact confirms the known concept that in allografted patients from a highly compatible donor, such as a matched sibling donor (MSD)—like the one of our patient—or a matched unrelated donor (MUD), post-transplant relapse is rarely attributable to loss of heterozygosity of class 1 HLA [21].

### Somatic mutations present at relapse

WES analysis of matched diagnosis and relapse samples revealed the presence of mutations occurring on *ZNF81* (ChrX:47916366–47916367; G > A; p.R574I; variant allele frequency (VAF) 44.23), *CTSC* (Chr11:88294204–88294205; T > C; p.N398S; VAF 41.82), and *ZGLP1* (Chr19:10305443–10305444; G > A; p.P215L; VAF 36.51) genes (Table 1). *ZNF81* is a member of the zinc finger gene family and encodes a protein that likely functions as a transcription factor, and germline mutations in this gene cause an X-linked form of intellectual disability (MRX45). *CTSC* encodes a member of the peptidase C1 family and lysosomal cysteine proteinase that appears to be a central coordinator for activation of many serine proteinases in cells of the immune system. Defects in the encoded protein have been shown to be a cause of the autosomal recessive disorder Papillon-Lefevre syndrome. *ZGLP1* encodes a transcriptional regulator that plays a key role in germ cell development. However, as further explained in the discussion section, none of these mutated genes show a potential role in oncogenesis.

**Table 1:** The molecular somatic alterations found at relapse are listed. The human genome GRCh38/hg38 was used as reference

Chromosome	Position	Reference base	Variant base	Gene	Variant (codon)	Variant (protein)	AA change*	Mutation type	Variant type*	Oncoscore
ChrX	47916369–47916367	G	T	*ZNF81*	AGA- > ATA	R574I	NonSynonymous	SNV	Somatic	5.267
Chr11	88294204–88294205	T	C	*CTSC*	AAC- > AGC	N398S	NonSynonymous	SNV	Somatic	15.841
Chr19	10305443–10305444	G	A	*ZGLP1*	CCG- > CTG	P215L	NonSynonymous	SNV	Somatic	0

### Fusion genes

**Fig. 3:**
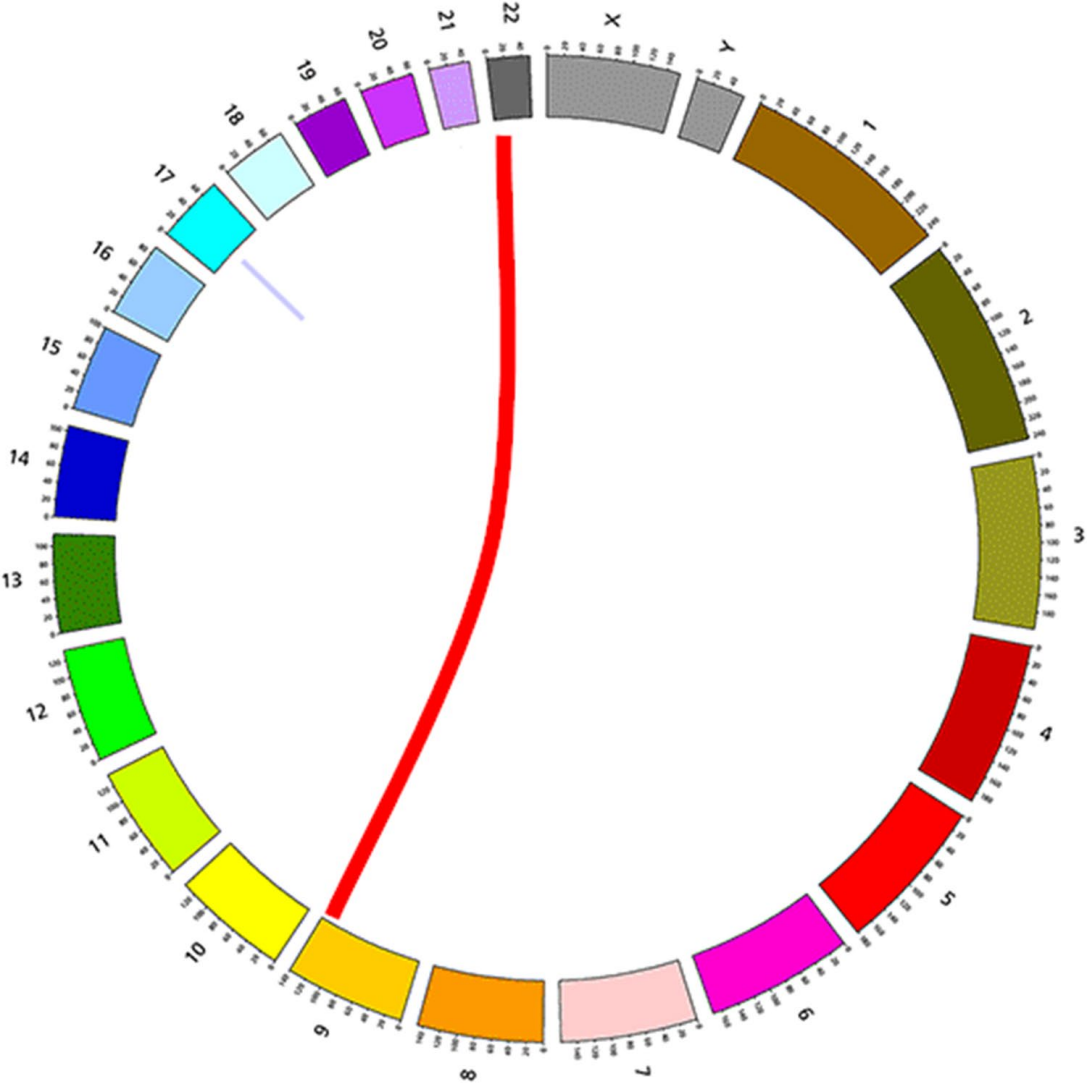
Fusion genes present at diagnosis. The red line indicates *BCR::ABL1* fusion gene, while the lavender line indicates *KANSL1::ARL17A* fusion gene

RNA sequencing analysis performed on the diagnosis sample showed the presence of two fusion genes: *BCR::ABL1* and *KANSL1::ARL17A* (*KANSARL)* (Fig. 3 and Table 2). *BCR::ABL1* is the pathognomonic fusion gene found in CML, while *KANSL1::ARL17A* is a chimeric gene resulted from the fusion between the *KANSL1* and *ARL17A* genes [22]. Unfortunately, we were not able to perform the same analysis on the relapse sample, due to bad quality of sample.

**Table 2:** The fusion genes present at diagnosis are listed. The human genome GRCh38/hg38 was used as reference

Chr1	Chr2	Gene1	Gene2	Position1	Position2	Mutation type	Coverage
Chr17	Chr17	KANSL	ARL17A	46094559–46094701	46352464–46352930	Fusion gene	13
Chr9	Chr22	ABL1	BCR	130713880–130714455	23292540–23292638	Fusion gene	39

## Discussion

CML relapses after allogeneic HSCT performed in CP occur after a median time of 9–15 months, even though a precise cut-off time to define a relapse as “late” does not exist [23, 24]. Few cases of CML relapses occurring many years after HSCT are described, and, to our knowledge, only 3 are documented at or after 20 years (precisely 20, 24, and 25 years). However, in none of these reports, the possible cause of leukemia recurrence has been investigated [9, 11, 25]. This fact may be due to the unavailability of the sample at diagnosis, which must have been kept in very good condition of viability for many years.

The patient presented in this report underwent allogeneic HSCT before the introduction of TKI and his CML relapsed many years later. A comparison of WES between relapse and diagnoses revealed a small number of somatic mutations appearing at relapse, involving 3 genes: *ZNF81*, *CTSC*, and *ZGLP1*. Using the OncoScore instrument [26], a novel, Internet-based tool to assess the oncogenic potential of genes, it is unlikely that these 3 somatic mutations are involved in the causation of relapse. Indeed, only Oncoscore values of at least 21.09 are considered indicative of a potential role in oncogenesis and none of these 3 mutations has such a value, amounting respectively to 5.267, 15.841, and 0.

The presence of such a little number of somatic mutations is in line with the hypothesis that CML relapses could be determined by a subpopulation of quiescent leukemic stem cells (LSC), which remain silent for many years and, thanks to their slow metabolism, are able to avoid the proapoptotic effect of different therapeutic agents, including traditional chemotherapy and radiotherapy, like the ones used in the patient for the conditioning regimen before transplantation [27]. These LSC show a very limited tendency towards the accumulation of somatic mutations and for this reason the mutational burden of *BCR::ABL1*-positive cells at the time of relapse can be very similar to that of diagnosis and rarely present drug resistance or tendency to disease progression [28]. In addition, this patient was never exposed to TKI, and therefore, the selective pressure operating during the 20 years post-transplant was aimed at circumventing GVL or other cellular processes, but not kinase inhibitors. This hypothesis is also coherent with the rapid and optimal response to TKI treatment, which let him achieve an MMR 6 months and a DMR 9 months after imatinib initiation.

Another interesting observation is the identification of the fusion transcript *KANSL1::ARL17A*. This is the first cancer predisposition fusion gene, familiarly inherited, specific to populations of European ancestry origin, identified in 30–52% of the samples from North Americans cancer patients affected by glioblastomas, prostate, breast, lung cancer, and lymphomas. This fusion gene was found, as a germline alteration, in 28.9% of the population of European ancestry origin, differently from the individuals from Asia or Africa, where it is rare or absent [22]. Both *ARL1* and *KANSL1* genes are located on the reverse strand of the chromosome 17q21.31. *KANSL1* encodes an evolutionarily conserved nuclear protein, a subunit of MLL1 and NSL1 complexes that is involved in histone H4 acetylation and p53 Lys120 acetylation [29]. KANSL1 is a microtubule-associated protein that localizes to the spindle poles and in the pericentriolar region during mitosis, contributing to microtubule assembly and stabilization, ensuring faithful chromosome segregation during mitosis. It is also known that knockdown of KANSL proteins leads to marked and terminal mitotic defects, associated with cancer or microdeletion syndrome [30]. *ARL17A* gene encodes a protein of the ARF family that is involved in multiple regulatory pathways relevant to human carcinogenesis [31]. The fusion peptide encoded by the *KANSL1::ARL17A* fusion transcript lacks some functional domains, and therefore, cancer patients expressing *KANSL1::ARL17A* display reduced activities of the histone acetyltransferase KAT8 and p53 [32–34]. Zhou et al. hypothesized that the reduction of these 2 proteins’ activities results in hypermutations in certain chromosomal regions of cancer cells and/or epigenetic changes that generate new read-through fusion transcripts [22]. Unfortunately, in our case, we were not able to demonstrate if the presence of *KANSL1::ARL17A* fusion gene was present only in somatic cells or also as a germline alteration. Moreover, we were not able to check whether the fusion gene was present in the donor. However, this finding could justify a condition of genomic instability which may be associated with the onset and possibly to the late relapse of his CML. Moreover, to our knowledge, this is the first case documenting the presence of the *KANSL1::ARL17A* fusion gene in a patient affected by CML.

Finally, this case is a rare example of a patient who successfully underwent a TKI discontinuation attempt after a post-HSCT relapse. According to the last US National Comprehensive Cancer Network (NCCN) guidelines, treatment-free remission (TFR) should be attempted in patients who have been on approved TKI therapy for at least 3 years and who have been in at least MR4 for at least 2 years [35]. Actually, our patient, whose TKI discontinuation was motivated by drug intolerance, stopped bosutinib after 35 months of total TKI treatment duration and after 26 months of stable DMR. Trials on TFR have usually excluded patients who had previously received an allogeneic HSCT. However, the possibility of a successful TKI discontinuation after achieving a new sustained DMR following a relapse occurring many years after transplantation should not be ruled out. Indeed, nowadays, we know that, in another clinical setting, a second successful discontinuation attempt can take place after the failure of a first attempt [36]. A possible biological explanation resides in the progressive exhaustion of quiescent LSC [37, 38]. Making a parallelism with the clinical history of our patient, we could speculate that, if his quiescent LSCs were the reason of his CML relapse almost 20 years after allogeneic HSCT, their eventual exhaustion after the time of a subsequent course of TKI therapy (never received before), leading to a sustained DMR (and even to stable undetectable transcript levels for 14 months before discontinuation) could support a subsequent stable TFR state. Actually, 5 months after bosutinib discontinuation, RT-PCR analysis in our patient turned positive, but he never experienced a molecular relapse (i.e., a loss of MMR) until the last follow-up, 43 months after TKI discontinuation.

These results confirm, as previously published by us and others [39–43], that residual *BCR::ABL1*^+^ cells persist, even after bone marrow transplantation and PCR negativity. Long-term monitoring of the patient is therefore necessary.

In summary, we presented here the case of a CML patient experiencing a relapse 20 years after allogeneic HSCT, emphasizing the need for long-term follow-up for allo-transplanted CML patients. Moreover, we provided some insights on the molecular mechanisms of his relapse, with a particular attention to the possible role of a new fusion gene, *KANSL1::ARL17A*, documented for the first time in this disease. Finally, this is a rare example of a successful TKI discontinuation attempt in a CML patient who previously underwent HSCT, had a late relapse, and re-achieved a sustained DMR thanks to TKI treatment.
